# *Conotrachelusterryerwini*, a majestic new species of Curculionidae (Molytinae, Conotrachelini) from Costa Rica

**DOI:** 10.3897/zookeys.1044.62722

**Published:** 2021-06-16

**Authors:** Robert S. Anderson

**Affiliations:** 1 Beaty Centre for Species Discovery, Canadian Museum of Nature, P.O. Box 3443, Station D, Ottawa, ON. K1P 6P4 Canada Canadian Museum of Nature Ottawa Canada

**Keywords:** Biodiversity, species discovery, tropical biology, weevil

## Abstract

A very large, new, and distinctive species of *Conotrachelus* Dejean is described from Área de Conservación Guanacaste, Costa Rica. *Conotrachelusterryerwini***sp. nov.** (type locality Volcan Orosi, Estación Biológica Maritza, Guanacaste, Costa Rica) is described and named in honor of Terry L. Erwin (1940–2020), famed carabidologist and biodiversity champion. This majestic species is easily distinguished by its large body size (15–20 mm) and extremely long rostrum (especially in females).

## Introduction

Many years ago while sorting weevils at the Instituto Nacional de Biodiversidad (INBio) in Santo Domingo, Costa Rica (now under the administration of the Museo Nacional de Costa Rica), a series of large specimens of a species of the genus *Conotrachelus* Dejean, 1835 were found. Most other species in the genus measure 5–8 mm in total body length but these specimens were gigantic by comparison at almost 20 mm in total body length with a rostrum in the females measuring almost twice the length of the elytra, meaning that from tip of rostrum (if extended anteriorly) to the end of the elytra, some females exceeded 40 mm. They clearly represented a new species but because of the diversity of the genus, they were set aside until the genus could be revised on at least a regional if not comprehensive manner. That revision is unlikely to happen soon, so the species is here described and named in honor of my recently deceased friend and colleague Terry L. Erwin.

*Conotrachelus* is a hyperdiverse genus limited to the Americas with more than 500 described species (O’Brien and Wibmer 1982; Wibmer and O’Brien 1986) and numerous undescribed species known (Pinzón-Navarro et al. 2010; Wolda et al. 1998). Champion (1904) treated 189 species from Central America, illustrated many of them, but only provided a key to informal groupings of the species (numbered by him) based on admittedly rather subjectively defined and variable sets of characters. *Conotrachelusterryerwini* keys to his group of species 6–16 although it does not appear to be close to any of them. Fiedler (1940) revised the South American members of the genus and added additional species in subsequent papers (Fielder 1944, 1952) and although I have not attempted to key *Conotrachelusterryerwini* in the Fiedler publications, none of his species appear close in body size. Species of the genus are associated with plant parts in a wide variety of plant families (Pinzón-Navarro et al. 2010). A small number of the tropical species are known as pests of avocados (Chamorro and Barclay 2018).

## Materials and methods

Specimens were examined using standard techniques for the study of dried insect specimens. Body length is measured from apex of elytra to anterior margin of pronotum (head and rostrum excluded). Classification follows Alonso-Zarazaga and Lyal (1999). Examined specimens are from or deposited in the following collections:

**ASUCOB**Arizona State University, Charles W. O’Brien Legacy Collection, Tempe, AZ, U.S.A.; E. Engasser, S. Lee;

**CMNC**Canadian Museum of Nature, Ottawa, Canada; F. Génier;

**JPPC** Jens Prena Private Collection, Rostock, Germany;

**MNCR**Museo Nacional de Costa Rica, San Jose, Costa Rica; M. Méndez Soto, A. Solis;

**NHMUK**The Natural History Museum, London, England; M.V.L. Barclay;

**USNM**United States National Museum, Washington D.C., U.S.A.; M. L. Chamorro.

## Taxonomy

### Conotrachelus terryerwini sp. nov.

Taxon classificationAnimaliaColeopteraCurculionidae

C17DA1B8-666F-5F0E-8302-97AFCAD5ECF2

http://zoobank.org/A7E931DE-45ED-4844-B61B-F660865C9B43

Figures 1–6

#### Material examined.

***Holotype*** male, labelled Costa Rica. Área de Conservación Guanacaste, Guanacaste Province, lado oeste Volcan Orosi, Estación Maritza, 27 Feb – 10 Mar 1992, F. Quesada, INBIOCRI000504110 (MNCR). ***Paratypes*** (n = 52): Costa Rica. Área de Conservación Guanacaste, Guanacaste Province, lado oeste Volcan Orosi, Estación Maritza, C. Cano, 28 Feb–10 Mar 1992, 10.95922, -85.49514, 580 m, INBIOCRI000750547 (1 male, CMNC), INBIOCRI000750544 (1 male, CMNC). Same data, INBIOCRI000750542 (1 male, MNCR), INBIOCRI000750545, INBIOCRI000750546, INBIOCRI000750585 (3 females, MNCR). Same data, K. Taylor, INBIOCRI000394982, INBIOCRI000394980 (2 females, CMNC), INBIOCRI000744294 (1 female, ASUCOB), INBIOCRI000394981 (1 female, MNCR). Same data, R. Guzman, INBIOCRI000441415 (1 male, ASUCOB), INBIOCRI000441416 (1 male, MNCR). Same data, M. Segura, INBIOCRI000789900 (1 female, CMNC), INBIOCRI000789899 (1 female, NHMUK), INBIOCRI000789901 (1 female, USNM), INBIOCRI000789902, INBIOCRI000789906, INBIOCRI000789907 (3 females, MNCR), INBIOCRI000789908 (1 male, NHMUK), INBIOCRI000789903 (1 male, USNM), INBIOCRI000789904, INBIOCRI000789905, INBIOCRI000789895 (3 males, MNCR). Same data, D. Garcia, INBIOCRI000536265 (1 female, MNCR). INBIOCRI000536266 (1 female, MNCR), INBIOCRI000536267 (1 female, MNCR). Same data, Feb 1992, P. Campos, INBIOCRI000888176 (1 female, CMNC), INBIOCRI000888177, INBIOCRI000888178 (2 females, MNCR). Same data, A. Gutierrez, 27 Feb – 10 Mar 1992, INBIOCRI000882467 (1 male, MNCR). Same data, R. Vargas, INBIOCRI000468740, INBIOCRI000468741 (2 males, MNCR), INBIOCRI000468742 (1 female, MNCR). Same data, D. Brenes, INBIOCRI000482368 (1 male, MNCR). Same data, M. Reyes, INBIOCRI000429924 (1 female, MNCR). Same data, S. Rojas, 26 Feb – 10 Mar 1992, INBIOCRI000707066 (1 male, MNCR). Same data, F. Araya, Feb 1992, INBIOCRI000737227 (1 male, MNCR), INBIOCRI000737088 (1 female, MNCR). Same data, M. Ortiz, 27 Feb 1992, INBIOCRI001727984, INBIOCRI001727985 (2 females, MNCR). Same data, May 1989, biodiversity survey, INBIOCRI000100349 (1 female, MNCR). Same data, K. Flores INBIOCRI000750809, INBIOCRI000750810, INBIOCRI000750806, INBIOCRI000750808, INBIOCRI000750543 (2 males, 3 females, MNCR). Same data, malaise trap, 1990, INBIOCRI000898487 (1 male, MNCR), INBIOCRI000037583 (1 female, MNCR). Same data, Aug 1990, Parataxonomos, INBIOCRI000258786 (1 male, MNCR). Parque Nacional Guanacaste, Estación Maritza, 600m, 15 Mar 1996, J. Prena (1 male, JPPC). Estación Mengo, SW side Volcan Cacao, 1100 m, 10°55’43”N, 85°28’10”W, Guanacaste National Park Biodiversity Survey, INBIOCRI001044881 (1 female, MNCR). Macio Miravalles, Estación Cabro Muco, 1100 m, 22 Jun – 8 Jul 2003, J. Azofieta, trampa de luz, INB0003731073 (1 female, MNCR). Same data, 27 Jun – 2 Jul 2003, L. D. Gutiérrez, INB0003740021 (1 male, MNCR).

**Figures 1–6.**
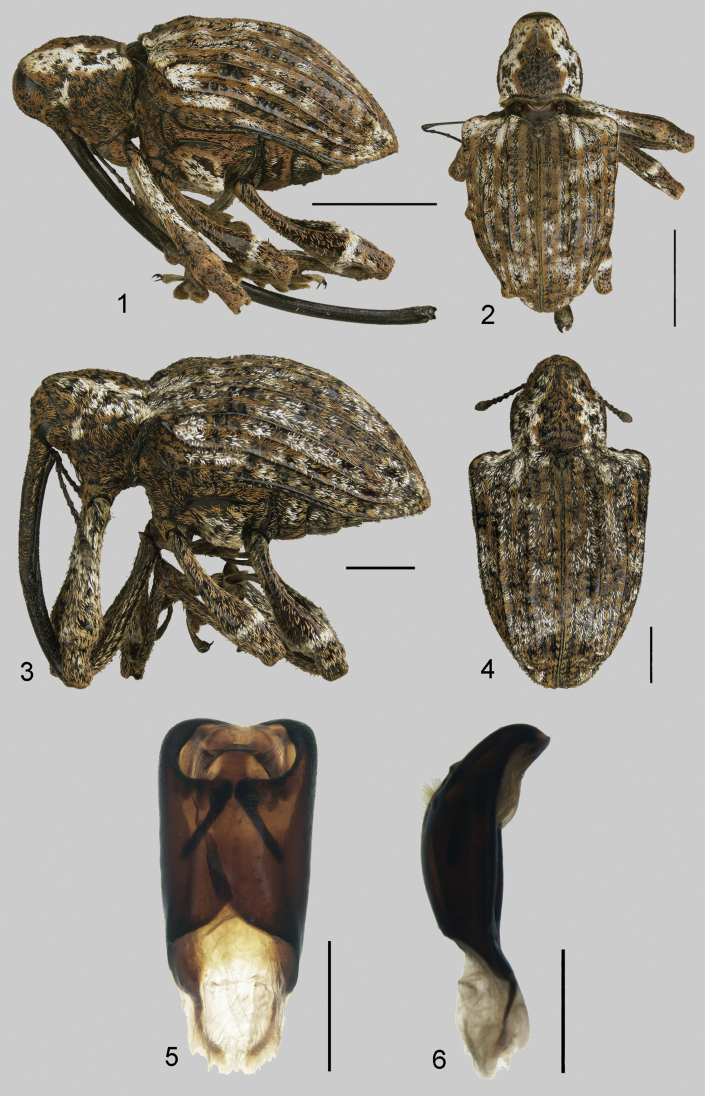
*Conotrachelusterryerwini***1** habitus, lateral, female (paratype INBioI000394980) **2** habitus, dorsal, female (paratype INBioI000394980) **3** habitus, lateral, male (paratype INBioI000750544) **4** habitus, dorsal, male (paratype INBioI000750544) **5** aedeagus, dorsal (paratype INBioI000750547) **6** aedeagus, lateral (paratype INBioI000750547). Scale bars: 1 mm (**5, 6**); 2 mm (**3, 4**); 5 mm (**1, 2**).

#### Diagnosis.

Large body size, 15–17 mm in males, 16–20 mm in females; body color black with scattered golden brown and white scales; rostrum extremely long in male and (especially) female, very slightly uniformly curved, ca. as long as elytra in male, 1.5–2.0× elytral length in female; pronotal postocular lobes large, completely covering eyes when rostrum in repose, discal area of pronotum projected anteriorly over the head; mesoscutellum strongly produced, bulbous; elytra with intervals 3, 5, 7, 8, 9 subcarinate, raised throughout various portions of length, all other intervals more or less flat; humerus strongly produced laterally, angulate, lateral margins of elytra convergent from humerus to apex; base of elytra ca. 2× width of base of pronotum; femoral teeth large, broad, distinct.

#### Description.

***Body*** black with scattered white and golden-brown scales, no visible suberect or erect setae. ***Head*** with large, shallow, separated punctures and sparse vestiture of golden-brown elongate scales; rostrum in male evenly curved, ca. as long as length of elytra, point of antennal insertion at ca. midlength, area distad of point of antennal insertion laterally irregularly, shallowly punctate, most punctures elongate, clearly separated, dorsally with scattered fine golden-brown scales throughout most of length; in female rostrum evenly curved, exceedingly long, ca. 1.5–2.0× length of elytra, point of antennal insertion slightly before midlength, area distad of point of antennal insertion very finely, sparsely, imperceptibly punctate, dorsally with scattered fine golden-brown scales only laterally towards base; apex of rostrum expanded laterally in both sexes, width greater than at point of antennal insertion; antennae with scape ca. as long as length funicle and club combined in male, slightly longer in female; third funicular article longer than second, second longer than first, 4–7 elongate, clavate, successively shorter, 4 slightly shorter than 1. ***Prothorax***: lateral margins subparallel to near apex, then constricted to apex; with large, shallow, individually indistinct punctures, surface somewhat rough due to raised posterior margins of punctures; median carina lacking; vestiture of scattered golden brown elongate scales medially, laterally with predominantly white scales in distinct lateral vitta on each side; in lateral view with postocular lobes very well developed, large, completely covering eyes when rostrum in repose, discal area produced anteriorly over the head. ***Elytra***: one-fourth to one-half longer than wide; sides strongly convergent to apex, base of elytra ca. 2× width of base of pronotum; humeri, distinct, acutely rounded; basal border sinuate mesad of humeri; intervals 3, 5, 7, 8, and 9 variously subcarinate to carinate, that of interval 5 evanescent basally, broadly rounded to subcarinate towards apex, that of interval 3 depressed subbasally and less strongly near declivity, slightly elevated just beyond base (elevation extended laterally to intervals 4 and 5), intervals otherwise flat or slightly rounded to carinate; serial punctures irregular in depth and size, not linearly arranged basally; with scattered vestiture of golden-brown and white elongate scales, white scales scattered over surface in small patches, otherwise mainly in a number of vague, diffuse transverse bands; elytra lacking scales in places, giving a somewhat mottled appearance; no suberect or erect setae present. Mesoscutellum bulbous. ***Ventral surface***: mesosternum flat posteriorly with rounded lobe, strongly sloping anteriorly. Abdominal ventrites sparsely and shallowly punctate; fifth ventrite without tubercles, not impressed; ventrites 1, 2, 5 with golden-brown scales moderately dense, ventrites 3 and 4 with scales much less dense; ventrites 1 and 2 equally convex in both sexes; ventrite 1 laterally ca. same length as ventrite 2, ventrites 3 and 4 subequal in length, each shorter than length of ventrite 2, ventrite 5 ca. same length as ventrites 3 and 4 combined. ***Legs***: pro-, meso-, and metafemoral tooth on inner margin large, broad, distinct; meso- and metafemur with band of condensed white scales on outer face at ca. apical 1/3 (at tooth), profemur with condensed white scales basally on inner and outer faces; tarsal claws widely divergent, each with short inner tooth. ***Male genitalia***: aedeagus 2× as long as wide and 4× as long as aedeagal struts; sides slightly divergent to near apex then strongly convergent to very slightly medially projected apex, no distinct apical process. Dorsal membranous area round, with basal median projection invading membranous area; lateral plates at confluence with basal projection with row of ca. 15 elongate hairs on each side of basal projection. Transfer apparatus complex (Fig. 5) with distinct, dark pair of convergent ‘wrench-like’ bars at middle. ***Female genitalia***: not examined.

#### Natural history.

Specimens of this species were first found by Pricilla Campos of Grano de Oro, Costa Rica, on the side of an unknown tree while learning entomology in the 1992 Female Parataxonomist course held in Estación Biológica Maritza, Area de Conservacion Guanacaste (ACG), Costa Rica. The species is almost exclusively known from specimens collected during that course around the Maritza station. The tree species remains unidentified but judging by the size and shape of the weevil, it is likely a seed predator in a large-fruited tree such as a species of *Manilkara* or *Pouteria* (Sapotaceae) or perhaps a species in the Lauraceae.

#### Comments.

This is the largest *Conotrachelus* known, far surpassing other species in length. Most species of the genus measure ca. 5–8 mm in length but large females of this species reach almost 2 cm. This species also has the longest rostrum of any *Conotrachelus* known, that of females up to 2 × as long as the elytral length and in lateral view (if directed posteriorly) extending between the legs and well beyond the apex of the elytra (Fig. 1). With the rostrum extended, a large female could measure more than 40 mm in total length.

#### Etymology.

It gives me great pleasure to name this species after my friend and colleague Terry L. Erwin (1940–2020). Terry completed his PhD at the University of Alberta where he studied under the legendary carabidologist George E. Ball, although some 20 years earlier than I. My close relationship with Terry dates back to the early 1990s when the famous tropical ecologist Dan Janzen asked Terry and me to convene some meetings to plan the Coleoptera component of the funding application for his All Taxon Biodiversity Inventory (ATBI) of the Área de Conservación Guanacaste in Costa Rica, and later, an extended proposal for the entire country of Costa Rica. We invited numerous Coleoptera taxonomists to participate in these planning meetings, to visit and study in the huge INBio collections, and to conduct field work. Although the project was never funded to the extent hoped for, many of us spent many hours together in the collections and associated hostel and developed life-long friendships. During subsequent years Terry would include an image of me, soaking and filthy from fieldwork in Panama, in many of his talks to illustrate that the profession of a tropical biologist was not easy or clean work. It was always the source of many good laughs and Terry always enjoyed showing it.

## Supplementary Material

XML Treatment for Conotrachelus terryerwini
